# Socioeconomic status and non-fatal injuries among Canadian adolescents: variations across SES and injury measures

**DOI:** 10.1186/1471-2458-5-132

**Published:** 2005-12-12

**Authors:** Beth K Potter, Kathy N Speechley, John J Koval, Iris A Gutmanis, M Karen Campbell, Douglas Manuel

**Affiliations:** 1 Department of Epidemiology and Community Medicine, University of Ottawa, Ottawa, Canada; 2 Department of Epidemiology and Biostatistics, University of Western Ontario, London, Canada; 3 Children's Health Research Institute, London, Canada; 4 Department of Paediatrics, University of Western Ontario, London, Canada; 5 Department of Obstetrics and Gynecology, University of Western Ontario, London, Canada; 6 Institute for Clinical Evaluative Sciences, Toronto, Canada; 7 Department of Public Health Sciences, University of Toronto, Toronto, Canada

## Abstract

**Background:**

While research to date has consistently demonstrated that socioeconomic status (SES) is inversely associated with injury mortality in both children and adults, findings have been less consistent for non-fatal injuries. The literature addressing SES and injury morbidity among adolescents has been particularly inconclusive. To explore potential explanations for these discrepant research findings, this study uniquely compared the relationship across different measures of SES and different causes of injury (recreation versus non-recreation injuries) within a sample of Canadian adolescents.

**Methods:**

The sample included adolescent participants (aged 12 to 19 years) in the Canadian 1996–1997 cross-sectional National Population Health Survey (n = 6967). Five SES measures (household income, two neighbourhood-level proxy measures, two parental indicators) were examined in relation to three injury outcomes (total, recreation, and non-recreation injuries) using multivariable logistic regression.

**Results:**

Among males, a clear relationship with injury was observed only for a parental SES index, which was positively associated with total and recreation injuries (odds ratios for the highest versus lowest SES category of 1.9 for total and 2.5 for recreation injuries). Among females, there was some evidence of a positive relationship between SES and injuries, particularly for a neighbourhood-level education measure with total and recreation injuries (odds ratios of 1.7 for total and 2.0 for recreation injuries).

**Conclusion:**

The results suggest that differences related to the measures of SES chosen and the causes of injury under study may both contribute to discrepancies in past research on SES and non-fatal injuries among adolescents. To clarify the potential SES-injury relationship among youth, the findings emphasize a need for a greater understanding of the meaning and relevance of different SES measures for adolescents, and for an exploration of the pathways through which SES may be related to injury risk.

## Background

An inverse socioeconomic gradient has been documented for numerous health outcomes, including adult and infant all-cause mortality and a large number of diseases, health conditions, and health status measures [e.g., [1]]. This may not generalize to adolescent populations, however. West argued, based mainly on the results of studies of social class and adolescent health from the United Kingdom, that there seemed to be a relative equalization of the SES-health gradient during adolescence, followed by a re-emergence in early adulthood [2]. A more complex picture has emerged from additional research, where inverse relationships have been observed inconsistently across measures of SES, health outcomes, and adolescent characteristics [e.g, [3-6]]. Social determinants of adolescent health, including SES, may be particularly relevant to the study of adolescent injuries; this area of research is the focus of our study.

Injuries are unique among health conditions because of their acute nature, and because they are, by definition, externally caused. This external causation emphasizes the potential relevance of the physical and social environment, including socioeconomic factors, in contributing to injury risk. Research to date has consistently demonstrated that SES is inversely associated with injury mortality in both children and adults, but findings have been less consistent for non-fatal injuries [7]. The literature addressing SES and injury morbidity among adolescents has been particularly inconclusive. While some studies including adolescents have reported an inverse SES-injury gradient [8-17], others have failed to observe such a gradient or have even found some evidence of a positive association between SES and injuries among youth [18-24].

One possible explanation for these discrepant research findings is that SES may be differentially related to injuries according to cause. For example, there is some evidence that SES may be positively related to sports and recreation injuries among children and youth [23-25], yet inversely associated with some types of traffic injuries [e.g., [10,15,17]] and intentional injuries [e.g., [10,26,27]]. This might be explained in part by differences in the intermediate factors or pathways that could account for an SES-injury relationship. For example, SES may be related to injuries in children and adolescents through such factors as parental care and supervision [28,29], the neighbourhood environment [30], and behaviours [24]. These characteristics likely relate differently to injury risk from different causes (for example, physical activity behaviours may be related to recreation injuries, while the neighbourhood environment may be important for traffic-related injuries).

A second and related possible explanation for the inconsistent findings in previous studies is the inclusion of different measures of SES. There is no consensus among researchers on how aspects of social position should be conceptualized, or how terms such as social class, SES, and socioeconomic position should be defined and used. For example, some authors distinguish between concepts of status or prestige and class or economic resources [31-33], while others distinguish among dimensions of social stratification based on access to various forms of capital (including material or financial capital, human capital, and social capital) [34-36]. Some also draw a distinction between social class itself, which is defined in terms of societal relationships, and the manifestation of social class in various dimensions of socioeconomic position [31]. In this paper we use the term SES to refer to all aspects of social standing collectively, by convention. There is also no agreement on how social position is best measured. Some nations (such as the United Kingdom) have a long tradition of viewing social stratification in terms of occupational class [37], while North American research has tended to place greater importance on income, education, and occupational status in relation to income and education [36]. However measured, though, it is generally recognized that different indicators of SES (such as household income, education, occupational status or class, and neighbourhood characteristics) tend to reflect distinct underlying aspects of social position, and thus may be expected to be differentially related to health outcomes [33].

Given the above complexities, it is not surprising that it is challenging to identify which of the various aspects of SES is most relevant for adolescent populations [5,38] and for injury outcomes. The possible pathways through which SES may be related to adolescent injury include behaviours (e.g., physical activity and risk behaviours) and environmental characteristics (e.g., potential hazards in the physical environment) that could be influenced, for example, by both status and access to material resources. Another possible contribution of SES measurement to inconsistent research findings in relation to adolescent injury is the notion that SES indicators may hold different meaning for different adolescent populations, depending on social and economic policies [10]; this may help to explain international differences in research findings.

A greater understanding of the nature of the relationship between SES and injuries among adolescents could help to inform injury prevention priorities and would contribute to a wider body of knowledge addressing the social foundations of adolescent health. Toward this understanding, we empirically examined possible explanations for the inconsistent results among previous studies. Specifically, this study uniquely aimed to identify whether the observed relationship between SES and adolescent injury differed according to: i) different measures of SES; or ii) different causes of injury (recreation injuries versus non-recreation injuries).

## Methods

### Sample and data sources

The sample included 6967 Canadian adolescents aged 12 to 19 years who participated in the 1996–1997 cross-sectional National Population Health Survey (NPHS) [39], which sampled household residents in all Canadian provinces based on a complex probability sampling design. Specific Research Ethics Board approval was not required for this investigation, which used non-identifiable secondary data.

Demographic and health information was collected for every member of each surveyed household. One member of each household was selected for a more detailed health interview conducted by telephone. The overall household response rate was approximately 83%; over 95% of selected individuals within households responded to the detailed interview [39]. Adolescents in the present study were those selected for a detailed health interview. For a small minority (238 or 3.4%), responses were provided through a proxy interview with another household member.

Socioeconomic information for other members of the household as well as information on total household income was provided by the adolescent or another household member; interviewers were instructed to obtain these data from a knowledgeable household member [40].

Additional socioeconomic data were obtained from the 1996 Canadian census, by linking adolescents' postal codes to census tabulations at the enumeration area (EA) level [41]. EAs, which covered all of Canada, consisted of approximately 125 to 440 households [42].

### Measures

#### Measures of SES

Of the five measures of SES (Table 1), three were included for the full adolescent sample: household income, neighbourhood income quintiles, and neighbourhood education quintiles. The other two SES measures, based on parental education and occupation, were included for a subgroup of the adolescents who were reported to be living with one or more parents.

**Table 1:** Description of SES Measures

SES Measure	Sample Included	Source of Information	Level of Measurement
Household income	Full sample	Income information reported by a household member	Individual
Neighbourhood income quintiles	Full sample	Census tabulations (EA level)	Neighbourhood
Neighbourhood education quintiles	Full sample	Census tabulations (EA level)	Neighbourhood
Parental Blishen SES Index	Adolescents living with parent/s	Parental occupation information reported by a household member	Individual
Parental education	Adolescents living with parent/s	Parental education information reported by a household member	Individual

Information on household income from all sources was collected from one member of each household. This information was used to create a 5-category variable that represented total household income adjusted for household size (categories were based roughly on multiples of low income cut-offs). Since fewer than 5% of adolescents were in the lowest category, the two lowest categories were combined, yielding a 4-category household income variable for the analyses.

The two neighbourhood-level indicators of SES were created using census tabulations at the EA level. Neighbourhood income quintiles were based on average household income adjusted for household size distribution and geographic area [41]. A neighbourhood education measure was based on the proportion of the EA population estimated to have completed secondary school. This variable was converted to quintiles within the study sample, stratified by region and by rural/urban status.

For adolescents reported to be living with parent/s, the highest value of SES among household members aged 25 years or older was considered to represent parental SES. This information was sometimes reported by the adolescent and sometimes by a household adult [39,40]. Parental education included 5 categories of attained educational credentials, from less than secondary school diploma to the completion of a university degree. Parental occupation information was used to determine values for the Canadian Blishen SES index, which is based on occupational prestige as well as education and income levels [43]. This variable was categorized into quartiles within the study sample, stratified by region and by rural/urban status.

#### Measures of injury

Adolescents were asked whether they had experienced any injuries in the previous 12 months that were serious enough to limit normal activities, and were given examples (such as a broken bone, a bad cut, or a poisoning) [39]. Those who reported that they had been injured were asked for details regarding the most serious injury they had experienced (the most serious injury was defined by the adolescent, rather than by using explicit criteria to measure injury severity). Adolescents were considered to have a recreation injury if they reported a most serious injury occurring at a place for recreation or sport (including a school playground). Those whose most serious injury occurred elsewhere were considered to have a non-recreation injury. The specific causes of injury (for example, "accidental fall", "being struck by a person or object", "physical assault") were also examined for all injured adolescents. Of the 725 adolescents whose most serious injury occurred at a place for recreation or sport, 14 were excluded from the sample because the reported cause of injury did not appear recreation-related (it was either a "car accident", or the cause was unknown); none of the 650 adolescents whose most serious injury occurred elsewhere were excluded on the basis of cause. Non-injured adolescents formed the comparison group for three injury outcomes (total injuries, recreation injuries, and non-recreation injuries).

### Data analysis

Following descriptive and bivariate analysis, multivariable logistic regression was used to examine the relationship between each SES measure and each injury outcome in separate models, adjusted for demographic variables that, based on the literature, may be independently associated with injury (age, rural/urban status, geographic region, and whether the adolescent was living with parent/s). Since preliminary analyses revealed gender differences in the observed relationships, all final analyses were conducted separately for males and females. Because of the large number of statistical tests, the interpretation of results focused on identifying consistent trends or gradients across levels of SES, rather than solely on statistical significance.

Since the NPHS used a complex sampling design to yield a representative sample, sampling weights were incorporated in all analyses. Variance estimates for descriptive and regression analyses were adjusted using bootstrap replicate weights to account for clustering [39]. In the bootstrapping approach, the variance of the estimator for a parameter is approximated using a large number of subsamples drawn from the same original sample [44,45]. Statistics Canada drew 500 such subsamples for the 1996–1997 NPHS, and provided researchers with a set of sampling weights (replicate weights) for each subsample [39]. To apply bootstrap variance estimation, we recalculated each parameter estimate using each set of replicate weights, and applied the variance of the replicate estimates to the statistical models.

The distribution of sampling weights had a wide range and was highly positively skewed, in part because there was a high variability in sampling fractions across provinces. This meant that a small number of adolescents with particularly high sampling weights had the potential to have a large influence on the parameter estimates in statistical models [46]. To reduce bias in the regression coefficients, therefore, influential outliers were identified using regression diagnostic statistics [47,48]. These influential outliers were excluded from the final weighted multivariable models. Because the aim was to compare results across SES and injury variables, observations excluded from one model were also excluded from comparison models. Fewer than 1.5% of observations were excluded in total, and all final weighted models were free of influential outliers. No observations were identified as influential in any of the unweighted multivariable models, and all excluded outliers had sampling weights that exceeded the 90^th ^percentile for the overall sample, strongly suggesting that the large sampling weight relative to other observations was the primary reason for their influence.

## Results

### Descriptive results

There were 6826 adolescents in the final sample, after excluding 141 participants due to missing data on key variables. For the analyses including parental SES measures, the sample was restricted to adolescents who reportedly lived with one or more parents (N = 5667), and a further 122 participants missing information on parental education were excluded (N = 5545). A high proportion of adolescents (21.2%) were missing household income information and a substantial proportion of those living with parent/s were missing Blishen SES index data (10.8%). Thus, rather than excluding these participants, "missing" was a separate category for these variables.

Activity-limiting injury was reported by 18.8% of adolescents (Table 2). While 10.9% reported experiencing recreation injury, 9.8% reported non-recreation injury. A higher proportion of males reported injury (21.0%), relative to females (16.5%).

**Table 2:** Sample Characteristics and Proportion Reporting Injury (weighted proportions)

	Total (N = 6826^1^)		Males (N = 3459^1^)		Females (N = 3367^1^)	
	%	95% CI	%	95% CI	%	95% CI
Aged 12–14 years	35.2	(33.2, 37.3)	34.8	(31.7, 37.8)	35.7	(32.5, 38.9)
Aged 15–17 years	39.6	(37.5, 41.7)	41.1	(38.1, 44.1)	38.0	(34.9, 41.0)
Aged 18–19 years	25.2	(23.3, 27.2)	24.1	(21.5, 26.8)	26.3	(23.4, 29.3)
Rural (vs.urban) residence	23.9	(21.7, 26.1)	22.7	(20.0, 25.5)	25.1	(22.0, 28.2)
Living with parents	84.0	(82.5, 85.6)	85.0	(82.7, 87.3)	83.0	(80.6, 85.5)

Household Income						
Lowest	13.3	(11.6, 15.0)	14.3	(11.7, 16.9)	12.2	(10.2, 14.3)
Lower-Middle	26.6	(24.4, 28.9)	25.7	(23.0, 28.4)	27.6	(24.4, 30.8)
Upper-Middle	27.7	(25.5, 29.8)	28.4	(25.6, 31.2)	26.9	(24.0, 29.8)
Highest	11.3	(9.8, 12.8)	11.4	(9.2, 13.5)	11.3	(9.2, 13.3)
Missing	21.1	(19.5, 22.70	20.3	(18.0, 22.5)	22.0	(19.6, 24.4)
Neighbourhood Income						
Lowest	17.0	(15.1, 18.8)	18.1	(15.6, 20.5)	15.8	(13.6, 18.0)
Lower-Middle	18.7	(16.6, 20.7)	17.6	(15.0, 20.1)	19.8	(16.7, 22.9)
Middle	19.7	(17.9, 21.5)	20.6	(17.9, 23.4)	18.8	(16.6, 20.9)
Upper-Middle	21.2	(19.1, 23.3)	21.6	(19.0, 24.2)	20.8	(17.8, 23.7)
Highest	23.5	(20.9, 26.1)	22.2	(19.3, 25.0)	24.9	(21.2, 28.5)
Neighbourhood Education						
Lowest	19.3	(17.2, 21.5)	18.4	(15.9, 20.8)	20.3	(17.3, 23.3)
Lower-Middle	19.3	(17.2, 21.4)	20.5	(17.7, 23.3)	18.0	(15.4, 20.6)
Middle	19.0	(17.3, 20.8)	19.7	(16.9, 22.5)	18.3	(15.8, 20.7)
Upper-Middle	20.3	(18.0, 22.6)	20.3	(17.4, 23.2)	20.3	(17.6, 23.0)
Highest	22.1	(20.1, 24.1)	21.1	(18.3, 23.9)	23.2	(20.4, 25.9)

Parental SES Index^2^						
Lowest	23.0	(20.9, 25.2)	22.4	(19.5, 25.3)	23.7	(20.5, 26.9)
Lower-Middle	21.5	(19.3, 23.7)	21.1	(18.3, 23.8)	22.0	(18.7, 25.3)
Upper-Middle	22.5	(20.5, 24.6)	22.1	(19.3, 24.9)	23.0	(20.0, 26.0)
Highest	22.1	(19.9, 24.3)	23.7	(20.3, 27.1)	20.3	(17.7, 23.0)
Missing	10.8	(9.0, 12.6)	10.7	(8.5, 12.8)	10.9	(8.5, 13.4)
Parental Education^2^						
<Secondary	10.3	(8.8, 11.9)	9.3	(7.4, 11.3)	11.4	(9.3, 13.6)
Secondary	15.2	(13.1, 17.2)	14.8	(12.4, 17.2)	15.6	(12.5, 18.7)
Some post-secondary	21.3	(19.2, 23.5)	21.2	(18.3, 24.1)	21.4	(18.4, 24.5)
College/trade diploma	29.0	(26.7, 31.3)	29.6	(26.2, 32.9)	28.5	(25.2, 31.7)
University degree	24.1	(22.0, 26.3)	25.1	(22.2, 28.0)	23.1	(20.2, 26.0)

Total Injury	18.8	(16.9, 20.6)	21.0	(18.4, 23.6)	16.5	(13.6, 19.3)
Recreation Injury^3^	10.9	(9.4, 12.5)	13.5	(11.1, 15.9)	8.3	(6.3, 10.3)
Non-Recreation Injury^4^	9.8	(8.4, 11.2)	9.9	(8.1, 11.8)	9.6	(7.2, 12.0)

CI = confidence interval

^1 ^Number in the unweighted sample who contributed to the weighted proportions shown (exceptions below)

^2 ^Denominator: unweighted n = 5545 who live with parents

^3 ^Denominator: unweighted n = 6176 (excludes 650 with non-recreation injury)

^4 ^Denominator: unweighted n = 6115 (excludes 711 with recreation injury)

### SES and injury

Among males, for the full sample SES indicators (household income, neighbourhood income quintiles, and neighbourhood education quintiles), some analyses yielded statistically significant differences in injury between specific categories of SES and the reference category (Table 3). None of these relationships represented clear gradients, though, and the direction of the association differed across measures of SES. Among males living with parent/s, there was little evidence for an association between parental education and injuries. The parental Blishen SES index, in contrast, was positively related to both total injuries and recreation injuries, although it was not clearly associated with non-recreation injuries.

**Table 3:** SES and Injuries Among Males: Multivariable Results^1^

All Adolescents	Total Injury (Unwtd N = 3430)		Recreation Injury (Unwtd N = 3079)		Non-Rec. Injury (Unwtd N = 2997)	
	OR^2^	95% CI	OR^2^	95% CI	OR^2^	95% CI
Household Income^3 ^(Reference=lowest)						
Lower-Middle	0.8	(0.5, 1.3)	0.9	(0.5, 1.6)	0.8	(0.5, 1.4)
Upper-Middle	1.2	(0.8, 1.9)	1.6	(0.9, 2.7)	0.9	(0.5, 1.5)
Highest	1.1	(0.7, 1.8)	1.3	(0.7, 2.6)	0.8	(0.4, 1.6)
Missing	0.9	(0.6, 1.4)	1.0	(0.6, 1.8)	0.8	(0.5, 1.4)

Neighbourhood Income^3 ^(Reference=lowest)						
Lower-Middle	*1.6	(1.0, 2.4)	1.5	(0.9, 2.6)	1.7	(0.9, 2.9)
Middle	1.2	(0.8, 1.8)	1.1	(0.6, 1.8)	1.4	(0.8, 2.5)
Upper-Middle	0.9	(0.6, 1.3)	0.9	(0.5, 1.5)	0.8	(0.5, 1.5)
Highest	1.3	(0.8, 2.0)	1.6	(0.9, 2.7)	0.8	(0.5, 1.5)

Neighbourhood Education^3 ^(Reference=lowest)						
Lower-Middle	*0.7	(0.5, 1.0)	*0.6	(0.4, 0.9)	0.9	(0.6, 1.5)
Middle	0.8	(0.6, 1.2)	0.6	(0.4, 1.0)	1.3	(0.7, 2.2)
Upper-Middle	1.0	(0.6, 1.5)	0.9	(0.5, 1.5)	1.1	(0.6, 1.9)
Highest	0.9	(0.6, 1.3)	0.9	(0.5, 1.5)	1.0	(0.6, 1.6)

Adolescents living with parent/s	Total Injury (Unwtd N = 2861)		Recreation Injury (Unwtd N = 2587)		Non-Rec. Injury (Unwtd N = 2488)	
	OR^4^	95% CI	OR^4^	95% CI	OR^4^	95% CI

Blishen SES Index^3 ^(Reference=lowest)						
Lower-Middle	1.3	(0.9, 2.0)	1.4	(0.8, 2.3)	1.3	(0.8, 2.2)
Upper-Middle	1.4	(0.9, 2.0)	*1.7	(1.1, 2.7)	1.0	(0.6, 1.6)
Highest	***1.9	(1.3, 2.8)	***2.5	(1.5, 4.0)	1.2	(0.7, 2.0)
Missing	0.7	(0.4, 1.2)	0.7	(0.4, 1.4)	0.7	(0.3, 1.4)

Parental Education^3 ^(Reference =< secondary)						
Secondary	0.8	(0.5, 1.3)	0.8	(0.4, 1.6)	0.8	(0.4, 1.4)
Some post-secondary	*0.5	(0.3, 0.9)	0.6	(0.3, 1.2)	**0.4	(0.2, 0.8)
College/trade diploma	1.0	(0.6, 1.6)	1.3	(0.7, 2.4)	0.7	(0.4, 1.3)
University Degree	0.8	(0.5, 1.4)	1.1	(0.6, 2.2)	*0.5	(0.3, 1.0)

CI = confidence interval; non-rec. = non-recreation; OR = odds ratio; SES = socioeconomic status; unwtd = unweighted

^1 ^Influential outliers excluded: 29 excluded from full sample analyses; 22 excluded from parent sample

^2 ^Odds ratios adjusted for age, rural/urban status, living arrangements, and geographic region

^3 ^Each socioeconomic status variable examined separately (not adjusted for other SES variables)

^4 ^Odds ratios adjusted for age, rural/urban status, and geographic region

* = p < 0.05; ** = p < 0.01; *** = p < 0.001 (relative to reference category)

Among females, there was evidence of a positive relationship between the full sample SES indicators and the injury outcomes (Table 4). The gradient was most apparent for neighbourhood education, particularly in relation to total and recreation injuries. There was also a statistically significant positive association for neighbourhood income and both total injuries and non-recreation injuries. Neither the parental Blishen SES index nor parental education was significantly related to injury among females living with parent/s.

**Table 4:** SES and Injuries Among Females: Multivariable Results^1^

All Adolescents	Total Injury (Unwtd N = 3333)		Recreation Injury (Unwtd N = 3068)		Non-Rec. Injury (Unwtd N = 3082)	
	OR^2^	95% CI	OR^2^	95% CI	OR^2^	95% CI
Household Income^3 ^(Reference=lowest)						
Lower-Middle	1.2	(0.7, 2.0)	1.2	(0.6, 2.6)	1.2	(0.6, 2.3)
Upper-Middle	1.1	(0.6, 1.7)	1.7	(0.8, 3.6)	0.7	(0.4, 1.2)
Highest	1.3	(0.7, 2.1)	1.6	(0.7, 3.4)	1.1	(0.5, 2.2)
Missing	0.8	(0.5, 1.2)	0.8	(0.4, 1.7)	0.7	(0.4, 1.3)

Neighbourhood Income^3 ^(Reference=lowest)						
Lower-Middle	1.3	(0.8, 2.0)	1.3	(0.7, 2.4)	1.2	(0.7, 2.3)
Middle	*1.6	(1.1, 2.5)	1.6	(0.9, 2.9)	1.6	(0.9, 2.9)
Upper-Middle	1.2	(0.8, 1.9)	1.1	(0.6, 2.0)	1.4	(0.8, 2.5)
Highest	**1.8	(1.2, 2.7)	1.8	(1.0, 3.2)	*1.7	(1.0, 3.0)

Neighbourhood Education^3 ^(Reference=lowest)						
Lower-Middle	1.1	(0.7, 1.6)	0.9	(0.5, 1.7)	1.2	(0.6, 2.1)
Middle	1.3	(0.8, 2.0)	1.5	(0.8, 2.5)	1.2	(0.6, 2.2)
Upper-Middle	1.3	(0.8, 2.1)	1.7	(1.0, 3.0)	1.0	(0.5, 2.0)
Highest	*1.7	(1.1, 2.5)	*2.0	(1.1, 3.4)	1.5	(0.8, 2.7)

Adolescents living with parent/s	Total Injury (Unwtd N = 2633)		Recreation Injury (Unwtd N = 2421)		Non-Rec. Injury (Unwtd N = 2420)	
	OR^4^	95% CI	OR^4^	95% CI	OR^4^	95% CI

Blishen SES Index^3 ^(Reference=lowest)						
Lower-Middle	1.1	(0.7, 1.7)	1.0	(0.6, 1.8)	1.2	(0.6, 2.1)
Upper-Middle	1.0	(0.6, 1.6)	1.0	(0.5, 1.8)	1.1	(0.6, 2.1)
Highest	1.3	(0.8, 2.0)	1.6	(0.9, 2.8)	1.0	(0.5, 1.9)
Missing	0.7	(0.4, 1.2)	0.8	(0.4, 1.6)	0.7	(0.3, 1.4)

Parental Education^3 ^(Reference =< secondary)						
Secondary	0.8	(0.5, 1.4)	0.8	(0.4, 1.7)	0.8	(0.4, 1.8)
Some post-secondary	1.0	(0.5, 1.7)	0.7	(0.3, 1.5)	1.2	(0.5, 2.8)
College/trade diploma	1.1	(0.6, 1.9)	1.0	(0.5, 2.0)	1.1	(0.5, 2.5)
University Degree	1.0	(0.6, 1.8)	0.9	(0.4, 1.8)	1.1	(0.5, 2.5)

CI = confidence interval; non-rec. = non-recreation; OR = odds ratio; SES = socioeconomic status; unwtd = unweighted

^1 ^Influential outliers excluded: 34 excluded from full sample analyses; 29 excluded from parent sample

^2 ^Odds ratios adjusted for age, rural/urban status, living arrangements, and geographic region

^3 ^Each socioeconomic status variable examined separately (not adjusted for other SES variables)

^4 ^Odds ratios adjusted for age, rural/urban status, and geographic region

* = p < 0.05; ** = p < 0.01; *** = p < 0.001 (relative to reference category)

## Discussion

### Summary

The results of this study suggest that differences related to the measures of SES chosen and the causes of injury under study may both contribute to discrepancies in past research on SES and non-fatal injuries among adolescents.

### Measures of SES

The most striking result in our study was the variability in the observed relationship between SES and injuries across different measures of SES. This is not entirely surprising, given the discrepant findings in previous studies. No consistent trends in the SES-injury association are apparent in the literature when comparing studies incorporating parental and household SES indicators based on parental occupation [10,20,22,24], parental education [18,23], household income [23], or adolescent perceptions of family affluence [22,24]. There is an intriguing pattern, though, whereby studies that have measured SES at the neighbourhood level have tended to observe an inverse relationship between SES and total (rather than cause-specific) non-fatal childhood or adolescent injuries [8,9,11-13,15-17], while studies that have focused on individual SES indicators have been less consistent, with several finding no relationship or a positive association between SES and injuries [20,22-24]. Interpretation of this pattern is limited by other differences between the two groups of studies, though. For example, studies incorporating area-based indicators of SES have tended to be ecological in nature, comparing population-based rates of health care use for injuries across neighbourhoods or regions [8,9,11-13,15-17], while studies relying on individual-level SES measures have tended to be based on injury self-reports among a defined sample of children or adolescents [20,22-24] (with some exceptions [10,18]). These two types of studies (ecological and individual) likely differ in terms of the distribution of included injuries according to severity and cause as well as in their measures of SES, which may help to explain the differing findings (particularly in view of evidence that the observed SES-injury relationship likely depends on the cause of injury [10,24,49]). The two groups of studies may also be subject to different types of potential biases (for example, recall bias in studies based on self-reports, and possible biases related to health care system factors in studies based on medical records) [50]. Further, our study allowed for a comparison of the observed SES-injury relationship across both neighbourhood and individual-level SES measures within the same sample, and the findings did not follow the literature trend; any evidence of an association between SES and injury was positive.

Potential explanations for the heterogeneity we observed across SES indicators include conceptual differences in the SES measures (i.e., in terms of the underlying constructs captured), differences in measurement error, or chance. While chance may account for the few statistically significant gradients observed, it seems unlikely to account for the variability across measures.

Regarding measurement error, the level of missing data was relatively high for household income and for the Blishen SES index. In previous analyses with this sample, adolescents who were missing household income information were less likely to be living in higher income neighbourhoods; thus, "missingness" was not completely random [51]. The heterogeneity of the results is still apparent, though, excluding the findings related to household income. The household and parental SES measures were sometimes reported by the adolescent and sometimes by a household adult. Neither SES levels nor correlations among the SES measures differed systematically based on the reporter, however [51].

It seems likely that conceptual differences among the measures of SES account for some of the heterogeneity in our findings. This is supported by pairwise correlations among these measures in the sample, which were generally below 0.6 [51]. The results do not allow us to define which aspects of socioeconomic position are captured by the different SES indicators, or which indicators are most salient for adolescents. For example, at the individual level, income may be seen as an indicator of material resources, while occupation and education may reflect both economics and status or prestige [32]. When parental indicators are incorporated, there is the added complexity that any influence of occupation and education is indirect. It is possible that the positive relationship we observed between the Blishen SES index and injuries among males is a reflection that this indicator is more meaningful for male youth, relative to other SES measures; indeed parental education may have lower relevance for some adolescents because it reflects the more distant past [52]. If additional research supports the positive association, potential explanatory pathways could be explored. For example, we could explore whether the SES index reflects social status among adolescents, and if so, whether social status in turn is associated with injury risk behaviours among male youth.

Correlations between individual-level and neighbourhood indicators of SES for both genders were typically below 0.30 in our study [51], suggesting that they were measuring different constructs, or that the neighbourhood indicators were poor proxies for individual SES. Even when used as proxies for individual status, area-based indicators of SES may capture area-level characteristics [53]; neighbourhood SES indicators may be particularly relevant for injuries due to their potential association with characteristics such as physical hazards [30]. This does not seem to be a plausible explanation, though, for our observation of a positive relationship between the neighbourhood indicators of SES and injuries among females. Again, potential intermediate factors could be explored in future research. For example, neighbourhood SES measures may be proxies for aspects of material resources among adolescents, and female youth with higher material wealth may have greater access to injury risk activities.

### Causes of injury

There was some evidence that the relationship with SES was different for recreation versus non-recreation injuries. For females, the positive SES-injury gradient was generally more consistent for recreation injuries. Similarly, among males, the parental Blishen SES index was positively related to recreation injuries but not non-recreation injuries. This positive association is consistent with previous research [23-25,49]. Behaviours such as overall levels of physical activity could be explored as possible intermediate factors in the relationship between SES and recreation injuries; such behaviours may be associated with recreation injury risk among adolescents [54,55], and adolescents with higher SES may be exposed to higher physical activity or sports participation levels [e.g., [56,57]].

Although SES was not consistently related to non-recreation injuries, the grouping together of all such injuries may have limited our ability to detect associations with more specific injury circumstances. Inverse relationships have been observed for SES and both traffic injuries [10,12,15] and intentional injuries [10,26,27] among children and adolescents.

### Limitations

An important contribution of this study was the comparison of several different measures of SES in relation to injuries within the same adolescent sample. There were some important limitations, however. First, the sample size was not sufficient to examine injury causes beyond the breakdown into recreation and non-recreation injuries. Non-recreation injuries may be relatively heterogeneous with respect to causes and their relationship to SES (for example, as described, the literature suggests that some types of traffic and intentional injuries may be inversely related to SES). Related to this, while physical assault and suicide attempt were included as possible causes of injury in the data captured for the survey, given the interview format and the self-reported nature of these data, it is possible that intentional injuries may have been under-reported, and thus underrepresented in the analysis (approximately 2% of the most serious injuries in the unweighted sample were reported to be intentional). The sample size was also insufficient to explore age differences within the adolescent sample.

It was not possible to determine the severity of adolescent injuries in this study. Previous research has yielded mixed results in terms of SES and injury severity for children and adolescents [11,12,24], suggesting that severity could play a role in contributing to inconsistent study findings. The more consistent inverse relationship that has been documented between SES and injury mortality also supports the importance of considering severity [7]. This is also complicated by the notion that injury severity is likely related to injury cause (for example, recreation and non-recreation injuries may differ in severity).

A further potential limitation was that our measures of injury relied on adolescent self-reports. Recall errors have been identified in surveys of childhood injury [50,58], and recall bias has been proposed as a potential explanation for positive relationships in studies of SES and injuries [23,59]. Although this warrants exploration in terms of the positive relationships observed, it seems unlikely that such bias explains the heterogeneity in our findings.

Additional indicators of SES that may be worth exploring in relation to adolescent injury include measures of adolescents' own social position [2,5], subjective evaluations of SES [e.g., [38]], and measures of deprivation [e.g., [60]]. Subjective SES measures were not available for this study; deprivation measures were excluded because they may not reflect a range of SES levels.

Finally, a multi-level modeling approach to the analysis of SES and injuries may help to distinguish between individual and neighbourhood-level associations. Our focus on separately exploring individual and neighbourhood SES indicators reflected our aim of identifying possible reasons for heterogeneity among the findings of previous studies. Our results reveal that additional development work is necessary at each level of analysis (individual and contextual), to further elucidate the meaning of indicators of adolescent SES and how they relate to injuries. Multi-level modeling approaches will be an important component of this work, and in particular for area-level SES measures, will be useful for distinguishing between aspects of the SES-injury association that are related to characteristics of neighbourhoods themselves, and those that are related to the aggregate characteristics of individuals living within neighbourhoods. In terms of neighbourhood characteristics, it may be informative to explore which particular aspects of the social and physical environment (for example, traffic hazards or crime) are contributors to injury risk, and how SES variables capture these characteristics. In light of recent attention being given to issues such as neighbourhood income distribution and health [61], it may also be worthwhile to explore whether the degree of SES homogeneity within neighbourhoods is related to injury, or whether the role of individual SES in contributing to injury risk is modified by neighbourhood characteristics. This may be especially interesting given that in our sample, household income and area-based income quintiles variables were only modestly correlated (Spearman rank correlation of approximately 0.3) [51].

## Conclusion

We found that the relationship between SES and injury varied depending on the choice of both measures of SES exposure and injury outcomes. The findings emphasize the importance of considering how different measures of SES may operate through potential pathways that may include behavioural, social, and environmental factors. Our results also highlight challenges in measuring different dimensions of socioeconomic position. A future priority will be to further develop our understanding of both the meaning and relevance of SES indicators and the theoretical basis for a potential SES-injury relationship among adolescents.

Despite the variability in our findings, any apparent association between SES and non-fatal adolescent injuries was positive. This has implications for injury prevention, since evidence of a positive association between SES and non-fatal adolescent injuries argues against focusing prevention efforts mainly on lower SES groups. A positive or null relationship with SES is consistent with some (but not all) previous studies in this area, and suggests a need for caution in generalizing the findings of inverse SES gradients for injury mortality to non-fatal injuries.

Our results support previous literature documenting the complex nature of the relationship between SES and adolescent health. The findings suggest that to understand the potential relationship between SES and non-fatal injuries among youth, key conceptual and measurement issues related to both SES and injury will need to be addressed. This may help to explain variability in injury risk among adolescents and may aid in identifying priority areas for injury prevention. Understanding the potential contribution of SES will also provide insight into the social context that underlies more proximate exposures to injury risk among adolescents.

## Competing interests

The author(s) declare that they have no competing interests.

## Authors' contributions

BKP participated in the design and coordination of the study, carried out all statistical analyses, and drafted the manuscript. KNS participated in the design, coordination, and supervision of the study, as well as revisions to the manuscript. JJK participated in the design, coordination, and supervision of the study and revisions to the manuscript, as well as providing statistical guidance. IAG, MKC, and DM participated in the design, coordination, and supervision of the study, as well as revisions to the manuscript. All authors read and approved the final manuscript.

## Pre-publication history

The pre-publication history for this paper can be accessed here:


